# Matrix stiffness modulates androgen response genes and chromatin state in prostate cancer

**DOI:** 10.1093/narcan/zcaf010

**Published:** 2025-03-20

**Authors:** Roosa Kaarijärvi, Heidi Kaljunen, Onni Niemi, Merja Räsänen, Ville Paakinaho, Kirsi Ketola

**Affiliations:** Institute of Biomedicine, University of Eastern Finland, 70210, Kuopio, Finland; Institute of Biomedicine, University of Eastern Finland, 70210, Kuopio, Finland; Institute of Biomedicine, University of Eastern Finland, 70210, Kuopio, Finland; Institute of Biomedicine, University of Eastern Finland, 70210, Kuopio, Finland; Institute of Biomedicine, University of Eastern Finland, 70210, Kuopio, Finland; Institute of Biomedicine, University of Eastern Finland, 70210, Kuopio, Finland

## Abstract

The interplay between the extracellular matrix (ECM) and prostate cancer has been shown to increase ECM stiffness, correlating with more aggressive disease forms. However, the impact of ECM stiffness on the androgen receptor (AR), a key target in prostate cancer treatment, remains elusive. Here, we investigated whether matrix stiffness influences prostate cancer progression, transcriptional regulation, chromatin state, and AR function in AR-positive prostate cancer cells under varying ECM stiffness conditions. We utilized ATAC-seq (assay for transposase-accessible chromatin with sequencing) and RNA sequencing under different ECM conditions, along with the SUC2 metastatic prostate adenocarcinoma patient dataset, to investigate the role of ECM stiffness in chromatin state and androgen response genes, as well as its impact on prostate cancer progression. Results demonstrated that increased ECM stiffness elevated the expression of genes related to proliferation and differentiation. In contrast, androgen response genes were most highly induced in soft ECM conditions. Integrating chromatin accessibility with transcriptomic data revealed that androgen response genes were more transcriptionally available in soft ECM conditions. Additionally, increased ECM stiffness upregulated genes associated with low overall survival in the SUC2 dataset. Taken together, our results indicate that high expression of hard matrix stiffness genes may promote prostate cancer progression, leading to more aggressive disease forms associated with poor survival.

## Graphical Abstract

**Graphical Abstract:**
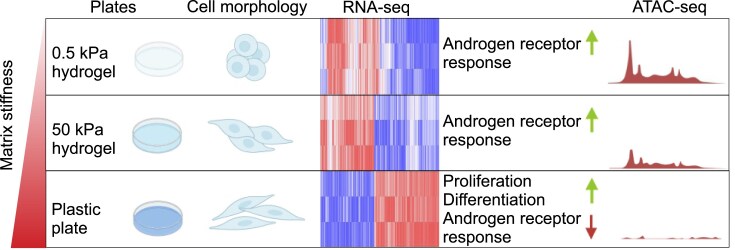

## Introduction

Prostate cancer is the second leading cause of cancer-related deaths in the Western world. The primary target for prostate cancer treatment is androgen receptor (AR); however, resistance to AR signaling-targeted therapies remains a significant challenge in the treatment of advanced prostate cancer. The effect of pressure and substrate stiffness on tumor development and progression has recently acquired increasing interest in cancer, including prostate cancer [1]. In particular, the extracellular matrix (ECM) stiffness within the tumor varies greatly during the disease progression. For example, in a comparative analysis of prostate tissue stiffness using shear wave elastography, it was shown that a benign prostate displays elastography values between 0 and 30 kPa, while in the tumors of advanced form of prostate cancer values go above 50 kPa [2]. In addition, there is a correlation between increased stiffness and higher Gleason score, suggesting that increased stiffness could be an indicator of prostate cancer disease progression [3, 4].

Cells sense and respond to the changes in ECM stiffness through mechanotransduction systems that translate the mechanical stimuli to biochemical signals to control cell proliferation, differentiation, and motility, among others [5–7]. Recently, increased matrix stiffness has been shown to activate mechanotransduction systems and subsequently signaling pathways related to proliferation and epithelial–mesenchymal transition (EMT) in AR-negative prostate cancer cells [1, 8]. However, the effect of matrix stiffness on transcriptional regulation, chromatin state, or the function of AR has not been explored before.

Here, we aimed at elaborating whether matrix stiffness plays a role in prostate cancer progression, transcriptional regulation, chromatin state, and AR function in various stiffness conditions from soft to hard ECM in AR-positive prostate cancer cells. The results indicated that increased matrix stiffness increased the expression of genes related to proliferation and differentiation. In contrast, androgen response genes showed the highest induction under soft ECM stiffness conditions. Furthermore, culturing cells in soft stiffness conditions correlated with increased chromatin openness and these open areas typically occurred at transcriptionally active promoters. Incorporation of chromatin accessibility with transcriptomic results revealed that androgen response genes were more available for transcription in soft matrix stiffness compared to hard stiffness culture conditions. Furthermore, increased matrix stiffness led to an upregulation of genes that correlate with low overall survival and that are often expressed in liver metastases, sites associated with poor prognosis in prostate cancer.

## Materials and methods

### Cell culture

Human prostate adenocarcinoma cell lines LNCaP and LNCaP C4-2B (referred to as C4-2B) were cultured in RPMI 1640 medium (Gibco) supplemented with 2 mM l-glutamine (Lonza), 10% fetal bovine serum (Gibco), and combination of 100 μg/ml streptomycin and 100 U/ml penicillin (Gibco Pen Strep).

### Hydrogel

The hydrogel culture dishes (Matrigen Softwell Petrisoft™ 100 mm Dish Easy Coat) and six-well plates (Softwell Easy Coat hydrogel six-well plates) containing polyacrylamide of two different stiffnesses (50 and 0.50 kPa) were purchased from Cell Guidance Systems. For activation, gels were coated with 0.2% rat collagen diluted in phosphate-buffered saline and incubated for roughly 1 h at 37°C before plating the cells.

### Cell imaging and morphology analysis

Cells were imaged after 1 week from plating with IncuCyte S3 Live-Cell Analysis System with 10× objective. For the aspect ratio calculations, the cell length and width were first measured in pixels using ImageJ software and then length-to-width ratio (i.e. aspect ratio) was calculated by dividing the length with the width.

### Quantitative real-time PCR and RNA-seq analysis

For RNA extraction, 250 000 cells were plated on six-well plates and grown for 1 week before sample collection. For quantitative polymerase chain reaction (qPCR), total RNA was extracted from cells using TRIzol (Invitrogen) reagent according to manufacturer’s guidelines. The extracted RNA was reverse transcribed to complementary DNA with Transcriptor First Strand cDNA Synthesis Kit (Roche) according to manufacturer’s instructions. Gene expression was analyzed using LightCycler 480 SYBR Green I Master (Roche), LightCycler 480 II (Roche) with 96-multiwell format, and 2^−ΔΔ^Ct method. Primers targeting AR (forward primer 5′-TTGGAGACTGCCAGGGAC-3′ and reverse primer 5′-TCAGGGGCGAAGTAGAGC-3′) and KLK3 (forward primer 5′-CACCCGAGCAGGTGCTTTTGC-3′ and reverse primer 5′-GGCAGGTGCTTGTGGCCTCTC-3′) were used.

For RNA sequencing (RNA-seq), Qiagen RNeasy Mini Kit was used to extract RNA, and RIN values were determined with a 2100 Bioanalyzer (Agilent). RNA-seq libraries were prepared using NEBNext Poly(A) mRNA Magnetic Isolation Module (New England Biolabs, E7490) and NEBNext Ultra II Directional RNA Library Prep with Sample Purification Beads Kit (New England Biolabs, E7765). Three biological replicate samples were sequenced with NextSeq 500 (75SE) at the EMBL Genomics Core Facility (Heidelberg, Germany). Sequenced raw reads were quality controlled, and the differential transcription was analyzed as described previously [9].

### Gene set enrichment analysis

Gene expression datasets were subjected to gene set enrichment analysis (GSEA) using GSEA software (v.4.3.3) from the Broad Institute (Massachusetts Institute of Technology) [10, 11]. The GSEA run was performed in weighted mode to identify significantly enriched pathways. Pathways enriched with a nominal *P*-value <.05 and false discovery rate (FDR) <0.25 were considered as significant.

### Western blot

For western blot, 250 000 cells were plated on six-well plates and grown for 1 week before sample collection. Cells were collected from plates with sodium dodecyl sulfate reagent containing proteinase inhibitor and total protein was extracted by sonication. Proteins were separated with sodium dodecyl sulfate–polyacrylamide gel electrophoresis and transferred to nitrocellulose membrane. Membranes were blocked with 5% non-fat dry milk for 1 h at room temperature followed by incubation with primary antibody overnight at 4°C. The next day, membranes were incubated for 1 h at room temperature with HRP-conjugated secondary antibodies. The proteins were detected with ECL reagent (Thermo Scientific™) by ChemiDoc MP Imaging System (Bio-Rad). The following antibodies were used: for GAPDH (FL-335), sc-25778 (Santa Cruz Biotechnology) with 1:5000 dilution; for MYBL2, b-Myb (F9W2M) #33056 (Cell Signaling Technology) with 1:1000 dilution; for AR, sc-7305 (Santa Cruz Biotechnology) with 1:400 dilution; and for secondary antibody, goat anti-rabbit IgG (H + L) (Invitrogen) or goat anti-mouse IgG (H + L) (Invitrogen) with 1:10 000 dilution.

### Luciferase reporter assay

LNCaP and C4-2B cells were seeded on six-well plates (300 000 cells per well) and grown for 24 h in transfection medium (RPMI 1640, 10% charcoal-stripped fetal bovine serum, 1% l-glutamine). Cells were transfected with pGL3-PSA5.8-LUC (received from Professor Jorma Palvimo, University of Eastern Finland, Finland [12]) and β-galactosidase expressing pCMVβ (Clontech) with Lipofectamine 3000 reagent (Promega). pGL3 empty vector was used as a negative control. After 24 h of transfection, cells were treated with 10 nM of synthetic androgen R1881 or ethanol for 24 h. To measure luciferase activity, samples were pipetted on an opaque 96-well plate and luciferase reagent (Promega) was added followed by measurement with a plate reader (Luminoskan Ascent, Thermo Fisher Scientific). For β-galactosidase, samples were pipetted on a clear 96-well plate and β-galactosidase reagent [1 mM MgCl2, 50 mM beta-mercaptoethanol, 1 mg/ml ONPG, 0.1 M sodium phosphate buffer, pH 7] was added and followed by measurement with a plate reader (Multiskan, Thermo Fisher Scientific) at 405 nm. Luciferase activity was determined by normalizing with β-galactosidase.

### ATAC-seq

For assay for transposase-accessible chromatin with sequencing (ATAC-seq), 2 million C4-2B cells were seeded onto 10-cm culture dishes (Nunc Cell Culture/Petri Dishes or Matrigen Softwell Petrisoft™ 100 mm Dish Easy Coat hydrogel plates) per culture. ATAC-seq was performed as described earlier [13]. For nuclei isolation, the cell pellets were resuspended in a concentration of 3 million cells per ml in Buffer A containing 15 mM Tris–HCl (pH 8), 15 mM NaCl, 60 mM KCl, 1 mM EDTA, 0.5 mM EGTA, and 0.5 mM spermidine (Sigma–Aldrich, S2626), supplemented with protease inhibitors (cOmplete™ Protease Inhibitor Cocktail, Roche). Cell suspension was diluted 1:1 ratio using Buffer A supplemented with 0.04% (w/v) IGEPAL (Sigma, #I8896). Samples were incubated on ice for 10 min, washed once with Buffer A without IGEPAL, and two times with ATAC resuspension buffer (10 mM NaCl, 10 mM Tris–HCl, 3 mM MgCl_2_). 100 000 nuclei were subjected to Tn5 transposition reaction using 2.5 μl TDE1 from Nextera DNA Library Prep Kit (Illumina, #FC-121-1030). After adding the transposition reaction mix, the samples were incubated for 45 min at 37°C with 800 rpm shaking, followed by DNA purification using Monarch PCR & DNA Cleanup Kit (New England Biolabs, #T1030) according to manufacturer’s instructions. DNA fragments were amplified using PCR and samples were barcoded using published primers. Amplified fragments were size selected (150–800 bp) using SPRIselect beads (Beckman Coulter, #B23318). Analysis of library quality was done with an Agilent 2100 Bioanalyzer using High Sensitivity DNA Analysis Kit (Agilent, #5067-4626). Two biological replicate samples were sequenced with Illumina NextSeq 500 (40PE) at the EMBL Genomics Core Facility (Heidelberg, Germany).

### ATAC-seq data analysis

Read quality filtering and alignment to hg38 genome was performed using Bowtie2 [14] using default settings. Downstream data analysis was performed using HOMER [15]. ATAC-seq peaks were called with findPeaks with style factor, FDR < 0.01 and >6-fold over local background. ENCODE hg38 blacklist regions were filtered from the peak list. Differential ATAC-seq peaks were defined with getDifferentialPeaksReplicates.pl (FDR < 0.1, log_2_FC > 0.5) between specified treatments (unchanged, UN; increased, UP; decreased, DN). Aggregate plots and heatmaps were generated with 10- or 20-bp bins surrounding ±1 kb area around the center of the peak. All plots were normalized to 10 million mapped reads and further to local tag density, tags per bp per site, whereas box plots represented log_2_ tag counts. Statistical significance in the box plots was determined using one-way analysis of variance (ANOVA) with Bonferroni post-hoc test. Box plots were generated using the Tukey method. Enrichment of sites to genomic elements was performed with annotatePeaks.pl. *De novo* motif searches were performed using findMotifsGenome.pl with the following parameters: 200 bp peak size window, strings with two mismatches, binomial distribution to score motif *P*-values, and 50 000 background regions. The results from the motif searches were displayed as heatmaps representing % of sites with motif. Only motifs that had 20% or higher enrichment in one of the regions (UN, UP, DN) were included in the heatmaps. Motif heatmaps were generated using hierarchical clustering with the Euclidean distance. ATAC-seq signal enrichment at androgen responsive gene sets was performed with annotatePeaks.pl. List of genes in the corresponding gene set was used with either tss of the genes or the closest ATAC-seq peak. Statistical significance was determined using one-way ANOVA with Bonferroni post-hoc test.

### *In silico* analyses

Median RNA-seq RNA expression values and clinicopathological data from metastatic prostate adenocarcinoma tumors (SU2C/PCF Dream Team, PNAS 2019) were analyzed using cBioPortal [16, 17].

## Results

### Prostate cancer cells lose their canonical elongated phenotype in soft matrix stiffness conditions

**Figure 1.**
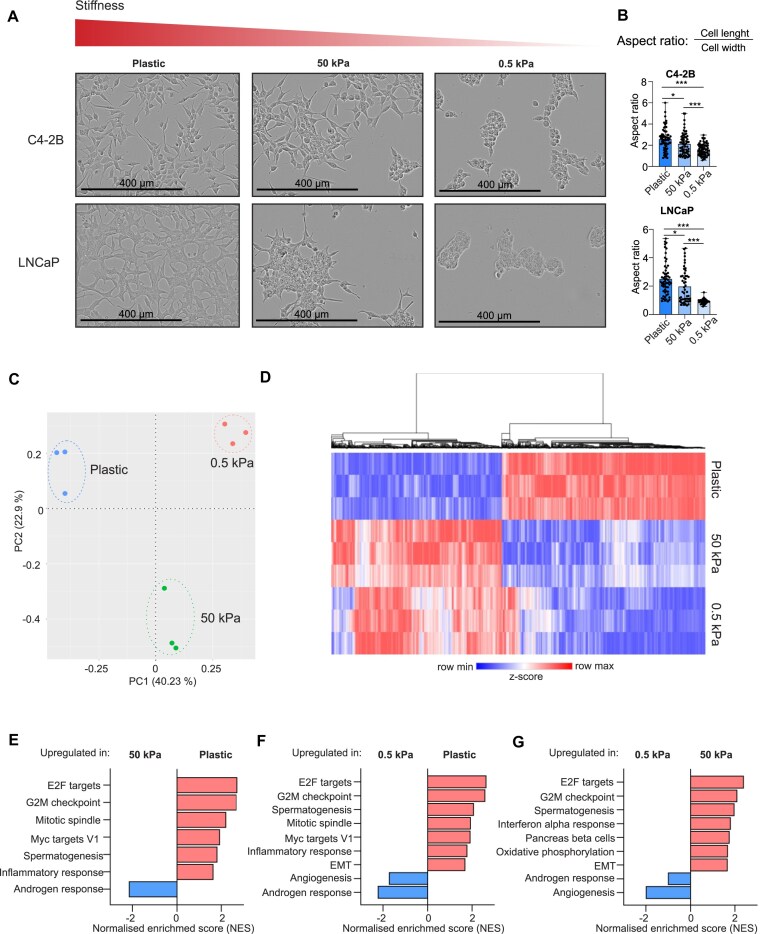
Matrix stiffness affects the cell morphology and transcriptional activity in LNCaP and C4-2B prostate cancer cells. (**A**) The IncuCyte live-cell imaging analysis shows the altered morphology of LNCaP and C4-2B cells after the cells were grown for 1 week on plastic, 50 kPa hydrogel, or 0.5 kPa hydrogel plates. Increasing matrix stiffness results in better adherence to the culture dish surface and canonical elongated morphology, while in soft 0.5 kPa hydrogel the cells appear to form grape-like clusters covering less surface area available. (**B**) A morphology analysis quantifying the aspect ratio (length-to-width ratio) conducted using ImageJ shows that cells grown in low stiffness (hydrogels) have significantly lower aspect ratio when compared to cells grown in high stiffness (plastic). The cells grown in plastic plates thus have more elongated shape, while cells grown in hydrogels are more rounded. Statistical significance is calculated with Student’s *t*-test. ***P < .001; **P < .01; *P < .05. (**C**) Principal component analysis (PCA) of RNA-seq of C4-2B cells grown on plastic, 50 kPa, and 0.5 kPa plates (*n* = 3) shows formation of distinct, matrix stiffness-dependent cell clusters. (**D**) Heatmap showing the top 10 000 differentially expressed genes (DEGs) between plastic and 50 kPa hydrogel (*P*-value <.001, fold change > |0.2|). Normalized read counts were used to count *z*-score and genes were hierarchically clustered using average linkage. Heatmap was created with Morpheus (https://software.broadinstitute.org/morpheus). (**E**) GSEA of hallmarks between plastic and 50 kPa pressure. High stiffness promotes expression of genes related to E2F targets, G2M checkpoint, mitotic spindle, Myc targets V1, spermatogenesis, and inflammatory response and downregulates genes related to androgen response. (**F**) GSEA of hallmarks between plastic and 0.5 kPa pressure. High stiffness promotes expression of genes related to E2F targets, G2M checkpoint, spermatogenesis, mitotic spindle, Myc targets V1, inflammatory response, and EMT and downregulates genes related to angiogenesis and androgen response. (**G**) GSEA of hallmarks between 50 and 0.5 kPa pressure. High stiffness promotes expression of genes related to E2F targets, G2M checkpoint, spermatogenesis, interferon alpha response, pancreas beta cells, oxidative phosphorylation, and EMT and downregulates genes related to angiogenesis and androgen response.

To study how ECM stiffness affects cell growth or the morphological phenotype of prostate cancer cells, androgen-sensitive cell line LNCaP and its metastatic derivative C4-2B were cultured on three different ECM stiffness conditions varying from 10 000 kPa of the conventional plastic plates to the stiff 50 kPa hydrogel plates, representing the stiffness of approximately early-stage prostate cancer tumors, and soft 0.5 kPa hydrogel plates. After a 1-week culture in these different ECM stiffness conditions, the cells’ morphology and growth were evaluated using the IncuCyte live-cell imaging system (Fig. 1A).

The results revealed that both cell lines exhibited typical two-dimensional monolayer growth pattern on hard plastic plates occupying the surface of the plate well bottom fairly evenly (Fig. 1A, left panel). The cells also displayed their canonical elongated cell morphology. However, when the stiffness of the growth matrix was reduced, a clear change in the cell morphology was observed: on 50 kPa hydrogels, the cells appeared to have a reduced ability to adhere to the well surface with cells stacking together instead of forming monolayers (Fig. 1A, middle panel). In addition, there was a minor shift to a slightly rounder cell morphology, especially in LNCaP cells. When the cells were cultured in the soft 0.5 kPa hydrogels, both cell lines lacked the canonical elongated cell morphology, forming more three-dimensional, grape-like cell clusters (Fig. 1A, right panel). The cells also appeared to adhere to the well bottom poorer as they covered less of the surface area available. To quantify the changes in cell morphology, aspect ratio (length-to-width ratio) of the cells was calculated. Values close to 1 represent a round cell and increased aspect ratios are seen in the elongated cells. Based on this analysis, cells grown on 0.5 kPa hydrogel have significantly lower aspect ratios (aspect ratio of 1.6 on C4-2B and 0.9 on LNCaP cells) when compared to cells grown on 50 kPa hydrogels (aspect ratio of 2) or in plastic (aspect ratio of 2.5) (Fig. 1B). This indicates that the cells in soft matrix have more rounded morphology, while with increasing stiffness the cells adopt a more elongated shape.

Taken together, both the cellular morphological phenotype and overall growth pattern of prostate cancer cells are influenced by the ECM stiffness.

### Soft matrix culture conditions promote the expression of androgen response genes, whereas hard matrix induces EMT gene sets

Next, we aimed at evaluating whether cellular gene expression is regulated by the matrix stiffness as the cells displayed loss of the canonical elongated morphology in soft matrix conditions. Since C4-2B cells appeared more adapted to grow on soft 0.5 kPa hydrogels, we investigated the potential transcriptional changes associated with this adaptation in C4-2B cells. First, we studied the overall difference of gene expression between the cells grown on plastic, stiff (50 kPa), or soft (0.5 kPa) hydrogels using RNA-seq. The PCA of the RNA-seq data revealed that C4-2B cells grown on plastic, 50 kPa hydrogel, and 0.5 kPa hydrogel grouped into their own distinct clusters (Fig. 1C), indicating that matrix stiffness induces transcriptional differences.

Next, we explored the specific differences in gene expression by comparing DEGs between the cells grown on plastic and 50 kPa hydrogel or 0.5 kPa hydrogel, and between 50 kPa hydrogel and 0.5 kPa hydrogel. The top 10 000 DEGs between plastic and 50 kPa (*P*-value <.001, fold change > |0.2|) were utilized in creating a heatmap showing that several genes were upregulated in C4-2B cells grown on plastic when compared to both 50 and 0.5 kPa hydrogels (Fig. 1D). Correspondingly, genes downregulated on plastic were upregulated at 50 and 0.5 kPa matrix stiffness. Overall, as expected, the most significant difference in the gene expression was observed between plastic and 0.5 kPa, while the gene expression in 50 kPa represented the average between the two.

To gain more insights into the matrix stiffness influenced pathways, GSEA on the RNA-seq data was performed. The GSEA revealed that several gene sets related to proliferation and cell cycle were significantly upregulated (positive normalized enrichment scores) in cells grown on plastic when compared to cells grown on 50 kPa hydrogel (Fig. 1E). These gene sets included E2F targets, G2M checkpoint, mitotic spindle, and Myc targets, and also genes related to spermatogenesis and inflammatory response. A similar trend was observed when comparing plastic to 0.5 kPa, including upregulation of gene sets related to EMT (Fig. 1F). EMT-related gene sets were also upregulated when 50 kPa hydrogel data were compared to those of 0.5 kPa hydrogel (Fig. 1G). Moreover, enrichment of gene sets upregulated in metastasis, stemness, and EMT was observed in response to increasing stiffness (Supplementary Fig. S1A–C). Furthermore, the gene set consisting of YAP-upregulated genes in YAP-dependent cancers described by Pearson *et al.* [18] was enriched in hard matrix stiffness alongside genes related to Rho GTPase activation. Both Rho GTPases and YAP are known mediators of changes in ECM stiffness [18, 19].

Surprisingly, androgen response genes were downregulated in cells grown on plastic when compared to cells grown on 50 or 0.5 kPa hydrogel (Fig. 1E and F, and Supplementary Fig. S1A and B.). The expression of androgen response genes was also lower in cells grown on 50 kPa hydrogel when compared to cells grown on 0.5 kPa hydrogel, suggesting that AR activity is reduced upon increased matrix stiffness. Lower stiffness appeared also to result in upregulation of core histone genes (Supplementary Fig. S1A and B; HDMs demethylate histones and HATs acetylate histones), suggesting changes in chromatin structure.

Finally, we compared using our RNA-seq data the top 100 up- and downregulated DEGs (Supplementary Table S1) between the three stiffness values to discover possible overlapping genes. Interestingly, five genes (*PBK*, *PCLAF*, *MYBL2*, *MCM10*, and *PIF1*) were among the most downregulated genes in each comparison (Supplementary Fig. S1D and E). MYBL2 has been shown to be important factor in prostate cancer progression as a very recent study introduces it as a driver of prostate cancer plasticity, while an earlier study reported that MYBL2 is upregulated in castration-resistant prostate cancer (CRPC) [20, 21]. We confirmed the decreased expression of MYBL2 in lower stiffness also at protein level using immunoblotting (Supplementary Fig. S1F).

In conclusion, increased ECM stiffness promotes the expression of gene sets related to proliferation, stemness, metastasis, and EMT, whereas androgen response gene sets and histone modification-related genes are enriched in soft matrix conditions. More specifically, androgen response-related genes appear to be upregulated in lower matrix stiffness.

### Soft ECM conditions promote chromatin openness on promoter regions

Our RNA-seq revealed that matrix stiffness affects the transcription by modulating the expression of several genes related to proliferation, cell cycle progression, histone modifications, and androgen response. Therefore, we were interested to see whether chromatin accessibility is also influenced. To our knowledge, the effect of matrix stiffness on chromatin accessibility has not been studied previously in prostate cancer cells.

**Figure 2.**
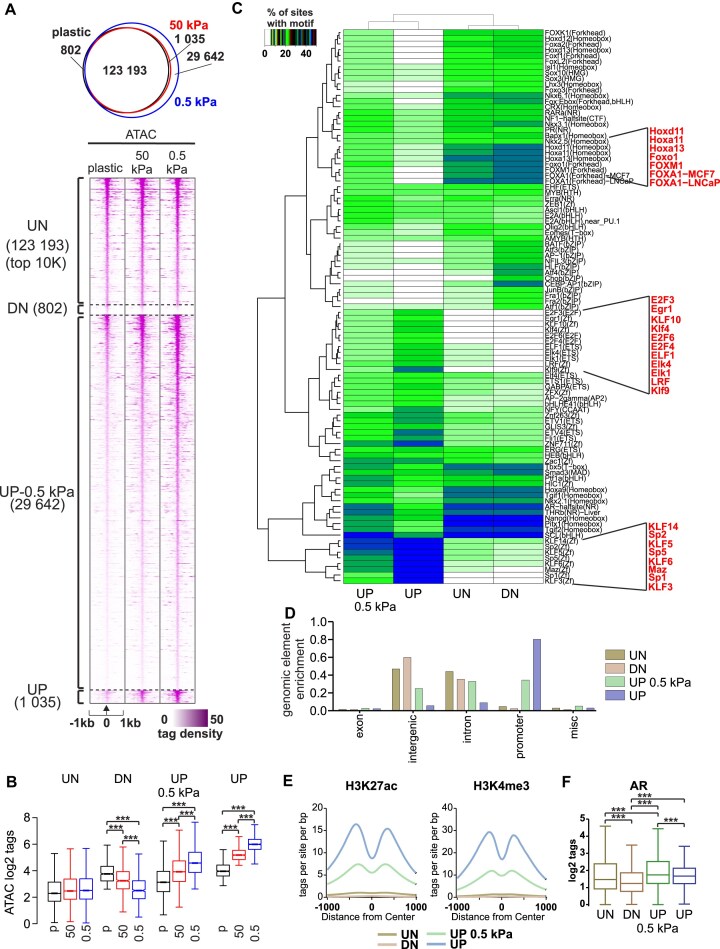
Chromatin openness increases in promoter regions in response to soft ECM. (**A**) ATAC-seq profiles of C4-2B cells grown in 1 week on plastic, 50 kPa hydrogel, and 0.5 kPa hydrogel plates (*n* = 2). 123 193 regions remained unchanged (UN); 802 regions were closed (DN) on 50 and 0.5 kPa when compared to plastic, 29 642 regions were opened on 0.5 kPa (UP-0.5 kPa), and 1035 regions were opened (UP) on both 0.5 and 50 kPa when compared to plastic. (**B**) Box plots of ATAC log_2_ tag density at unchanged (UN), closed (DN), opened at 0.5 kPa (UP-0.5 kPa), and opened (UP) at both 50 and 0.5 kPa sites. Statistical significance is calculated with one-way ANOVA with Bonferroni post-hoc test. ***P < .001. (**C**) *De novo* motif enrichment at indicated regions. Enrichment is displayed as a heatmap representing % of sites with motif. The scale is displayed on the side of the heatmap with white–green–blue indicating the prevalence of enrichment. Relevant motifs are highlighted in red. (**D**) Peak annotation of genomic elements. The majority of unchanged (UN) and closed (DN) regions are in intergenic and intron areas, while opened (UP) areas on both 0.5 and 50 kPa are in promoter regions. (**E**) Active histone modifications detected in indicated regions. Opened areas (UP) in both 50 and 0.5 kPa are susceptible to contain H3K27ac and H3K4me3. (**F**) AR binding is enriched at indicated regions in C4-2B cells. Statistical significance is calculated with one-way ANOVA with Bonferroni post-hoc test. ***P < .001.

To assess chromatin accessibility, we performed ATAC-seq in C4-2B cells. Chromatin openness was compared between cells grown on plastic, stiff 50 kPa hydrogel, and soft 0.5 kPa hydrogel. A global analysis revealed that a majority of chromatin regions remained unchanged (UN), while 800 regions were decreased or closed (DN) on 50 and 0.5 kPa hydrogel samples when compared to plastic samples (Fig. 2A and B). Surprisingly, almost 30 000 regions were increased or opened in 0.5 kPa hydrogel samples when compared to both plastic and 50 kPa samples (UP-0.5 kPa), while further 1000 regions were more accessible in 50 and 0.5 kPa hydrogel samples when compared to plastic samples (UP). This suggests that chromatin accessibility is affected by changes in matrix stiffness, and more specifically, soft matrix, represented by 0.5 kPa hydrogel, promotes chromatin openness.

To obtain insight into the differences between the regions, we performed motif analyses. While FOX- and HOX-family motifs (e.g. FOXA1, FOXM1, HOXA11, HOXD11) were enriched at UN and DN regions, both UP regions were enriched with SP-, KLF-, E2F-, and ETS-family motifs (e.g. SP1, KLF4, E2F3, KLF5, ELK1) (Fig. 2C). The former transcription factors bind more to enhancers, while the latter transcription factors bind more to promoters. Thus, matrix stiffness has preferential effect on either enhancer- or promoter-bound transcription factors. Because of this, we examined the genomic element enrichment of the chromatin accessibility areas in soft matrix stiffness conditions (Fig. 2D). The enrichment was concordant with motif analyses with UN and DN regions being largely at intergenic and intron areas, while UP regions were highly enriched in promoters.

To further understand the regulators of chromatin openness, we analyzed whether the enriched motifs from ATAC-seq correlated with gene expression changes in our RNA-seq data (Supplementary Fig. S2A). First, we found that FOXM1 and HOXA11 expression levels are significantly downregulated in 0.5 and 50 kPa stiffness conditions compared to plastic, whereas KLF5 expression is upregulated in response to decreasing matrix stiffness. FOXM1 is a known major contributor to prostate cancer progression and its upregulation correlates with low disease-free survival [22], whereas KLF5 functions as a tumor suppressor and is downregulated in more advanced stages of cancer progression [23]. We then analyzed the expression of FOXM1 targets (FOXM1 signature [24]) using our RNA-seq data and found that these genes were also downregulated in 0.5 and 50 kPa stiffness conditions compared to plastic (Supplementary Fig. S2B). These results suggest that a network of several transcription factors contribute to the changes in chromatin accessibility.

Next, we analyzed the enrichment of publicly available active histone modification data to our ATAC-seq data. To that end, we utilized H3K27ac [25] (GSM2825930, GSM2825931) and H3K4me3 [26] (GSM984397) ChIP-seq data. We observed that the UP regions displayed high levels of H3K4me3 and H3K27ac, indicating that the regions represent active promoters (Fig. 2E). The enrichment of these modifications was noticeably lower in UN and DN chromatin areas when compared to the UP regions. Finally, publicly available AR ChIP-seq data [27] indicated that out of all the regions, AR shows the most prominent occupancy at UN and UP-0.5 kPa regions (Fig. 2F). Thus, AR is capable of binding to both enhancer and promoter sites and accessible chromatin in low stiffness could enhance the activity of AR.

In conclusion, low matrix stiffness appears to result in more open areas in promoter regions, and these regions are associated with active histone modifications, indicating their potentially high transcriptional activity.

### Soft matrix promotes chromatin openness in androgen response gene areas

**Figure 3.**
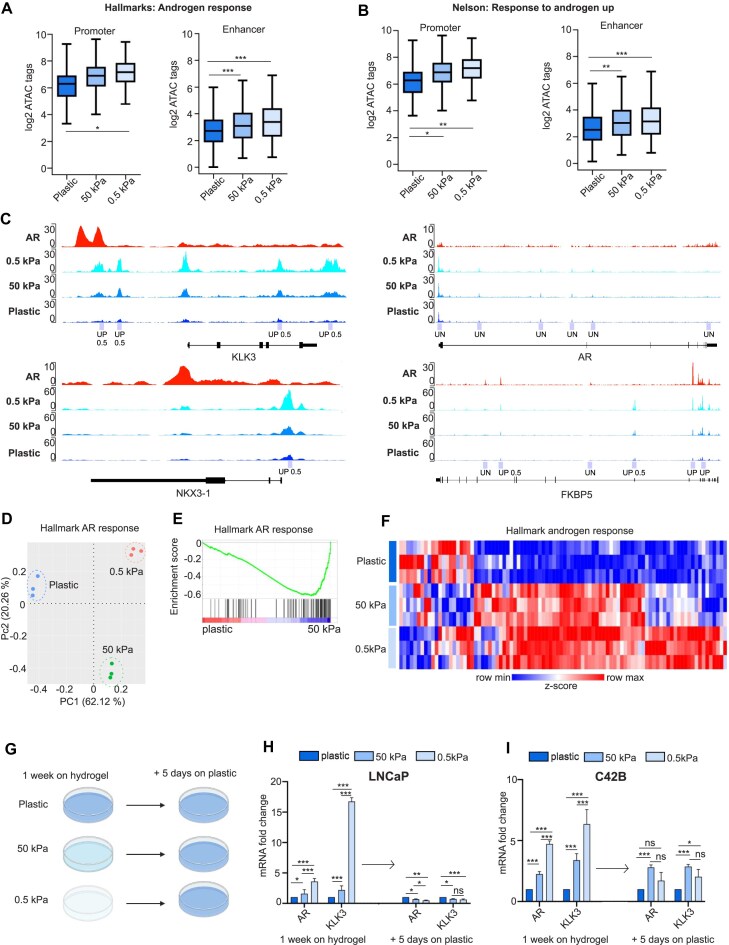
Chromatin is more open in androgen response gene areas in soft ECM. (**A**) Box plots of ATAC log_2_ tag density at promoter and enhancer regions of hallmarks: androgen response gene set showing that chromatin is more open in these areas in soft 0.5 kPa hydrogel. Statistical significance is calculated with one-way ANOVA with Bonferroni post-hoc test. ***P < .001; **P < .01; *P < .05. (**B**) Box plots of ATAC log_2_ tag density at promoter and enhancer regions of Nelson: response to androgen up gene set. Statistical significance is calculated with one-way ANOVA with Bonferroni post-hoc test. ***P < .001; **P < .01; *P < .05. (**C**) Visualization of the gene regions of AR and its target genes *KLK3*, *NKX3-1*, and *FKBP5*in 0.5 kPa, 50 kPa, and plastic. Peaks from AR ChIP data [20] are shown on the top and significantly accessible regions are marked below. The figures were created with the Integrative Genomics Viewer [21]. Genes of hallmark androgen response presented as (**D**) PCA plot, (**E**) enrichment, and (**F**) heatmap. Heatmap was created with Morpheus (https://software.broadinstitute.org/morpheus) and normalized read counts were used to count *z*-score and genes were hierarchically clustered using average linkage. (**G**) A visualization of the experiment setup used to analyze the reversal of changes induced by softer ECM. Cells were first cultured for 1 week on plastic, 50 kPa hydrogel, or 0.5 kPa hydrogel plates and then transferred to plastic plates for additional 5 days of growth. Created in BioRender (R. Kaarijärvi, 2025, https://BioRender.com/u54h207). The expression levels of AR and its target KLK3 are significantly upregulated in LNCaP cells after 1 week on 50 and 0.5 kPa hydrogel plates when compared to plastic and in turn both are reverted to basal levels after replating on plastic. (**I**) Similar results are obtained in C4-2B cells where the expression levels of AR and KLK3 are first upregulated when grown on hydrogels and then downregulated after replating on plastic.

Our observation that soft matrix stiffness promoted both the expression of androgen response genes and chromatin openness led us to investigate whether the chromatin accessibility at the promoter and enhancer regions of androgen response genes is altered. Analysis of the chromatin accessibility of the androgen response gene set genes showed significantly higher open chromatin in promoter regions on 0.5 kPa hydrogel when compared to plastic (Fig. 3A). Similar, but not significant, increase was seen with 50 kPa hydrogel. Additionally, significantly more open chromatin was observed at enhancer regions associated with androgen response genes in both 0.5 and 50 kPa hydrogels when compared to plastic. A similar effect was observed in “Nelson response to androgen up” gene set, where promoter and enhancer regions of androgen response genes were significantly more open in both 50 and 0.5 kPa hydrogels when compared to plastic (Fig. 3B). Furthermore, inspection of selected loci revealed increased chromatin accessibility in response to decreased matrix stiffness in AR gene locus, as well as in the regions of androgen response genes *KLK3* (encoding PSA), *NKX3-1*, and *FKBP5* (Fig. 3C).

Next, we took a closer look into RNA-seq data from C4-2B cells grown on either standard plastic culture plate or hydrogel plates (50 and 0.5 kPa) focusing specifically on the androgen response gene set (human gene set: HALLMARK_ANDROGEN_RESPONSE [28]). In a PCA plot of androgen receptor target genes, a similar formation of individual clusters depending on the growth matrix stiffness was observed as was seen before in the PCA analysis of the full transcriptome data (Fig. 3D). However, the protein levels of AR did not significantly increase (Supplementary Fig. S3A). As the GSEA showed a downregulation of androgen response genes on plastic (Fig. 3E), a heatmap of the genes was created and the expression of androgen response genes was compared between the different stiffness samples (Fig. 3F). As expected, in C4-2B cells grown on soft 0.5 kPa hydrogel, the expression levels of androgen response genes were highest, thus correlating with our ATAC-seq results where low matrix stiffness induced chromatin openness, specifically at promoters and enhancers of androgen response genes. Furthermore, we performed luciferase reporter assay of KLK3 promoter to study the AR binding in response to synthetic androgen R1881 (metribolone) and detected significant increase in AR activity on 0.5 kPa stiffness (Supplementary Fig. S3B).

### Soft matrix-induced androgen response is reversible

To determine whether soft matrix-induced androgen response is reversible, LNCaP and C4-2B cells were first cultured on plastic and both 50 and 0.5 kPa hydrogel plates for 1 week and then transferred to plastic culture plates for additional 5 days (Fig. 3G). The expression levels of AR and KLK3 were analyzed before and after transferring to plastic plates. After 1 week on 50 kPa hydrogel, a significant 2-fold increase in KLK3 and 1.5-fold increase in AR messenger RNA (mRNA) levels was detected in comparison to plastic in LNCaP cells, while a further 3.5-fold increase in AR and 16-fold increase in KLK3 mRNA levels was observed in cells grown on 0.5 kPa hydrogel. After transferring cells from hydrogels to plastic plates, the mRNA levels of both AR and KLK3 significantly decreased to 0.7-fold on 50 kPa and to ∼0.5-fold on 0.5 kPa hydrogel in LNCaP cells (Fig. 3H). A similar trend was seen in C4-2B cells grown on hydrogels, in which AR mRNA was increased 2.2-fold and KLK3 mRNA 3.3-fold on 50 kPa, and 4.7-fold and 6.3-fold on 0.5 kPa, respectively (Fig. 3I). Both AR and KLK3 mRNA expression levels returned closer to the basal plastic AR and KLK3 levels especially after replating C4-2B cells from 0.5 kPa to plastic. We also included AR-positive VCaP and 22RV1 cell lines in the experiment but did not observe any significant changes in the expression of KLK3 (Supplementary Fig. S3C).

In conclusion, based on our results the expression levels of AR and KLK3 increase in response to decreasing matrix stiffness and the phenomenon is reversible when replating the cells back to plastic.

### Genes upregulated in hard matrix conditions correlate with low overall survival

In the end, to determine whether soft or hard ECM stiffness gene signatures correlate with patient survival, we generated hard ECM stiffness gene set (“plastic signature”) from top 50 DEGs overexpressed in plastic samples compared to 0.5 kPa hydrogel samples. For 0.5 kPa hydrogel gene set (“0.5 kPa signature”), we selected top 50 most significantly overexpressed DEGs in 0.5 kPa hydrogel samples compared to plastic samples. First, we determined the expression levels of plastic signature genes in metastatic prostate adenocarcinoma patient dataset (SU2C [29, 30]). Data were organized based on tumor site.

**Figure 4.**
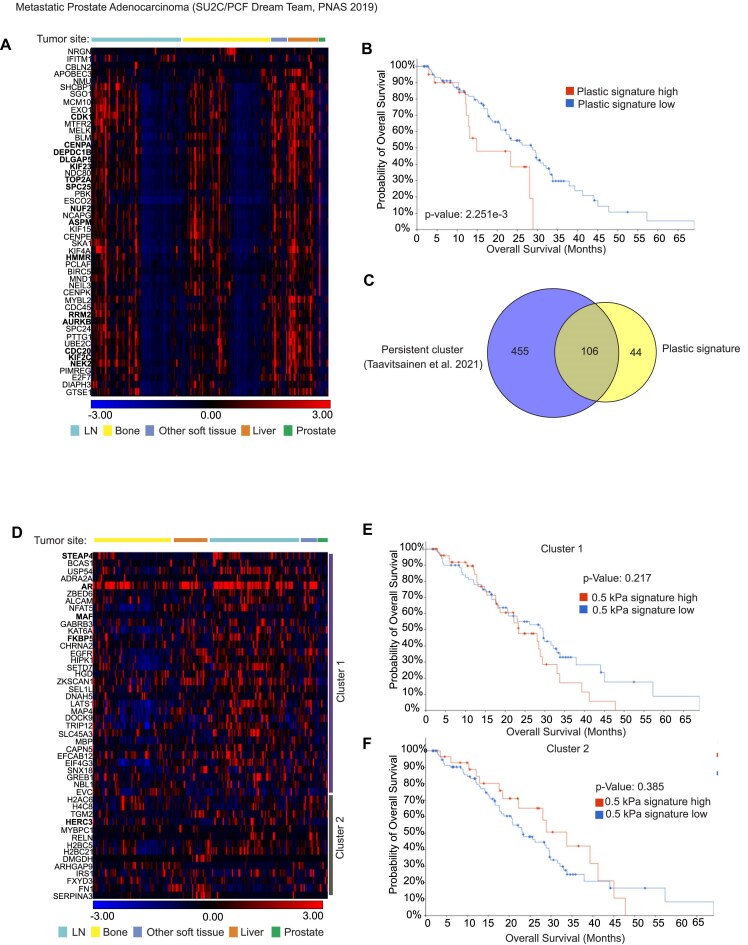
Upregulation of plastic signature genes correlates with low overall survival. (**A**) A heatmap showing the expression of plastic signature genes on metastatic patient tumor samples from lymph node, bone, other soft tissue, liver, and prostate. Upregulated FOXM1 target genes are highlighted in bold. (**B**) The overall survival of patients with high expression of plastic signature genes is low. (**C**) The plastic signature genes derived from our data overlap with genes belonging to persistent cluster described by Taavitsainen *et al.* [31]. (**D**) The expression of 0.5 kPa signature genes on metastatic patient tumor samples from lymph node, bone, other soft tissue, liver, and prostate. Genes are divided into two clusters with *k*-means clustering. Androgen response genes are highlighted in bold. There was no detectable effect on overall patient survival in either (**E**) cluster 1 or (**F**) cluster 2 from 0.5 kPa signature genes.

Interestingly, genes upregulated in “plastic signature” divided both lymph node patient tumors (blue) and bone metastasis patient tumors (yellow) into two groups, where in the first group plastic signature genes were upregulated and in the second group downregulated (Fig. 4A). Upregulation of plastic signature genes was also seen in tumors with liver metastases (Supplementary Fig. S4A). Furthermore, high expression of plastic signature genes correlated with low overall survival in prostate cancer patients (Fig. 4B).

As we detected an interesting pattern where plastic signature genes were upregulated in half of the lymph node and bone metastasis samples, we wanted to analyze in more detail whether there is a potential prognostic value of these gene sets in prostate cancer. A Venn diagram analysis indicated that out of the 150 plastic signature genes, 100 common genes were shared with the androgen deprivation therapy-persistent cell clusters (described by Taavitsainen *et al.* [31]), which have high regenerative potential with poor prognosis, thus supporting the notion that increased matrix stiffness associates with more aggressive form of prostate cancer (Fig. 4C). Earlier we showed that there was FOXM1 motif enrichment in DN regions (hydrogels versus plastic) based on our ATAC-seq data (Fig. 2C), and additionally a decrease in the expression of FOXM1 and its target genes in response to decreased ECM stiffness based on our RNA-seq data (Supplementary Fig. S2). Therefore, we wanted to find out whether FOXM1 signature is also seen among plastic signature genes and thus upregulated in patient metastatic tumor samples. Several FOXM1 targets were found upregulated as part of plastic signature in the patient samples (highlighted in bold in Fig. 4A), indicating that FOXM1 is a potential key contributor to the response in ECM stiffness as promoter of cancer progression.

Contrarily to plastic signature, the 0.5 kPa signature genes did not form as clear patient dividing patterns. Thus, the 0.5 kPa signature genes were divided into two clusters by using *k*-means clustering (Fig. 4D). There were no significant differences observed on overall survival between these two clusters with high and low 0.5 kPa signatures (Fig. 4E and F). Additionally, we studied whether the genes upregulated in 50 kPa when compared to 0.5 kPa also showed reduction in overall survival as in plastic upregulated genes (Supplementary Fig. S3B). Similarly to plastic signature, 50 kPa signature genes were mostly upregulated in liver metastases (Supplementary Fig. S3B) and after *k*-means clustering both clusters had lower overall survival when compared to patient samples with low expression of 50 kPa signature genes (Supplementary Fig. S4C).

In conclusion, these results indicate that high expression of hard matrix stiffness genes potentially promotes prostate cancer progression leading to more aggressive forms of the disease with poor survival rate.

## Discussion

In this study, we explored whether matrix stiffness plays a role in transcriptional regulation, chromatin state, or AR function in prostate cancer cells. The results revealed that genes related to proliferation and differentiation were upregulated in hard ECM stiffness conditions, whereas androgen response genes were induced in soft ECM stiffness conditions. In addition, the results indicated that chromatin was more open in soft matrix stiffness conditions, and these open areas typically occurred at active enhancers and promoters. Incorporation of ATAC-seq results with RNA-seq revealed that in soft matrix stiffness, androgen response genes were more available for transcription. Furthermore, hard matrix stiffness led to an upregulation of genes that correlated with low overall survival in prostate cancer patients and were correspondingly mostly found to be expressed in liver metastases.

Previously, it has been shown that an increased matrix stiffness leads to upregulated expression of estrogen receptor and activation of progesterone receptor [32–34]. To our knowledge, this is the first time the effect of matrix stiffness on androgen response and chromatin state is studied in prostate cancer. As induced AR signaling is an important driver of prostate cancer progression, and increased matrix stiffness is often connected to a more advanced disease, our findings showing that androgen response genes are specifically overexpressed in soft ECM conditions and rather downregulated in hard ECM conditions were surprising. It should be noted that upregulation of AR response was not observed in all AR-positive cell lines studied. One potential explanation could be in the expression of AR splice variant, but this would require further studies that expand beyond the scope of this study.

From our RNA-seq data, we detected MYBL2 and FOXM1 downregulation in low ECM stiffness. To our knowledge, neither of these proteins have been directly linked as mediators of ECM stiffness. MYBL2 overexpression has been shown to inhibit Hippo signaling by regulating expression of RACGAP1 leading to activation of YAP signaling in prostate cancer [21]. In addition to this, it has been shown that MYBL2 is a downstream effector of Akt/FOXM1 signaling in glioma [35]. A recent publication inspected the relationship between YAP signaling and AR and discovered that YAP can inhibit the transcriptional program of AR in AR-positive prostate cancer [36]. YAP was suggested to compete with AR binding to transcription factor TEAD, which diminishes the ability of AR to bind to its promoter and enhancer regions leading to reduced expression of AR markers and consequently slower proliferation of prostate cancer cells. YAP, on the other hand, has been linked to response in changes in matrix stiffness [37]. In our study, matrix stiffness was found to regulate chromatin accessibility, in particular at both promoters of AR regulated genes and enhancers linked to them. More specifically, in 50 and 0.5 kPa hydrogel samples, 1000 new open regions were observed when compared to plastic, and these open areas were mainly focused on promoter regions. Additionally, regions of androgen response genes were more open on soft matrix stiffness. Combining this observation with the ability of YAP to localize to nucleus to inhibit AR signaling in response to increased stiffness, this could explain why AR is more active on soft stiffness. More comprehensive studies are needed to confirm whether potential FOXM1/MYBL2/YAP pathway is responsible for regulating AR activity in response to changes in ECM stiffness in prostate cancer.

Even though we detected diminished androgen response upon increasing matrix stiffness, which in context of prostate cancer could be beneficial, we also noticed increased expression of proliferation, stemness, invasion, and EMT-related genes. Our results coincide with previous studies where increased stiffness was linked to metastasis and EMT in AR-negative (AR-independent) prostate cancer cell lines, thus supporting the importance of matrix stiffness in the progression of prostate cancer [1, 8]. Based on our findings, increased ECM stiffness could force the cancer to become androgen independent while activating other pro-tumorigenic pathways. Loss of AR signaling and AR independence in prostate cancer is one of the major drivers of CRPC and its more lethal and aggressive subtype, treatment-induced neuroendocrine prostate cancer (t-NEPC). Several AR-suppressed genes have been reported, which drive the development of t-NEPC, including BRN2, SOX2, and DPYSL5 [38–40].

Furthermore, genes upregulated on plastic related to lower overall survival rate and aggressive liver metastases, whereas genes upregulated in soft matrix, 0.5 kPa, conditions did not have similar significant effects on overall survival. Plastic upregulated genes had significant overlap with genes described belonging to “persistent cluster’’ by Taavitsainen *et al.* [31], which related to aggressive type of prostate cancer with regenerative abilities. This suggests that hard ECM contributes to disease aggressiveness of prostate cancer despite driving down the androgen receptor signaling.

We also detected the plasticity of the effects of changes in ECM stiffness, as replating cells grown on soft surfaces onto plastic plates brought the androgen response gene expression back to basal levels seen in plastic samples. Cells grown on plastic plates were able to spread better with mesenchymal-like morphology alongside increased expression of invasion markers, suggesting high stiffness driving the cancer metastasis. However, as the cancer breaks from the primary tumor into bloodstream or lymphatic system, this can cause a change in the pressure the cells experience. We detected a dramatic change in chromatin accessibility at a soft stiffness of 0.5 kPa when compared to plastic or 50 kPa; 30 000 new regions were opened or existing ones were significantly increased. Previous studies have reported that soft matrix leads to increased histone acetylation and decrease in histone methylation in liver stem cells and thus to more transcriptionally active, i.e. open, chromatin [41]. It could be speculated that as the soft matrix stiffness leads to increased chromatin accessibility, this helps the circulating tumor cells to adapt to the novel environment of their site of metastasis.

In conclusion, our results suggest that the function of androgen receptor is modulated by matrix stiffness. Soft stiffness promoted expression of genes regulated by androgen receptor and increased chromatin accessibility in both promoter and enhancer regions of these genes. Hard matrix stiffness regulated genes promoting prostate cancer progression and high expression of these genes that correlated with low overall survival in metastatic prostate adenocarcinoma patients.

## Supplementary Material

zcaf010_Supplemental_Files

## Data Availability

The cBioPortal for Cancer Genomics is an open-access portal (http://cbioportal.org) that enables interactive, exploratory analysis of large-scale cancer genomics data. ATAC-seq and RNA-seq data have been deposited to GEO (GEO accession: GSE272029).
