# Supplementary material for: Matrix stiffness modulates androgen response genes and chromatin state in prostate cancer
Source: NAR Cancer. 2025 Mar 20;7(1):zcaf010. doi: 10.1093/narcan/zcaf010 (PMC11923743; doi:10.1093/narcan/zcaf010)
Supplement: zcaf010_Supplemental_Files [file zcaf010_supplemental_files.zip › supfig1.pdf]

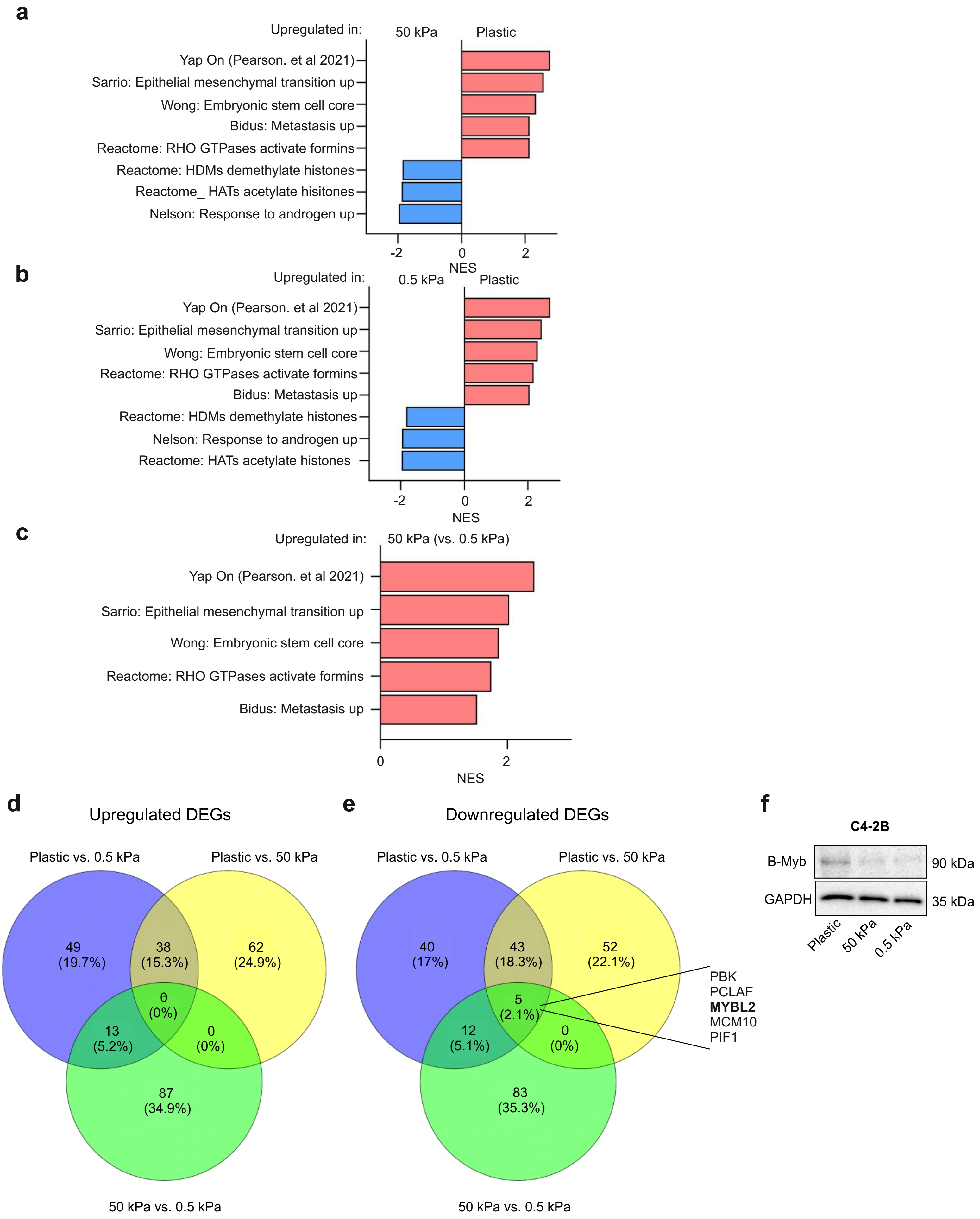

Supplementary Figure 1. a) Gene set enrichment analysis (GSEA) of curative gene sets between plastic and 50 kPa pressure. High stiffness promotes expression of genes related to active YAP signaling, epithelial mesenchymal transition, embryonic stem cell core, metastasis and RHO GTPase signaling and downregulates genes related to HDMs demethylates histones, HATs acetylate histones and androgen response. b) Gene set enrichment analysis (GSEA) of curative gene sets between plastic and 0.5 kPa pressure. High stiffness promotes expression of genes related to active YAP signaling, epithelial mesenchymal transition, embryonic stem cell core, metastasis and RHO GTPase signaling and downregulates genes related to HDMs demethylates histones, HATs acetylate histones and androgen response. c) GSEA analysis of curative gene sets between 50 kpa and 0.5 kPa pressure. High stiffness promotes expression of genes related to active YAP signaling, epithelial mesenchymal transition, embryonic stem cell core, metastasis and RHO GTPase signaling. d) Comparison of common upregulated DEGs between plastic vs. 0.5 kPa, plastic vs. 50 kPa and 50 kPa vs. 0.5 kPa e) Comparison of common downregulated DEGs between plastic vs. 0.5 kPa, plastic vs. 50 kPa and 50 kPa vs. 0.5 kPa f) Western blot shows downregulation of protein levels of B-Myb in lower stiffness (MYBL2)
