# Supplementary material for: Matrix stiffness modulates androgen response genes and chromatin state in prostate cancer
Source: NAR Cancer. 2025 Mar 20;7(1):zcaf010. doi: 10.1093/narcan/zcaf010 (PMC11923743; doi:10.1093/narcan/zcaf010)
Supplement: zcaf010_Supplemental_Files [file zcaf010_supplemental_files.zip › supfig2.pdf]

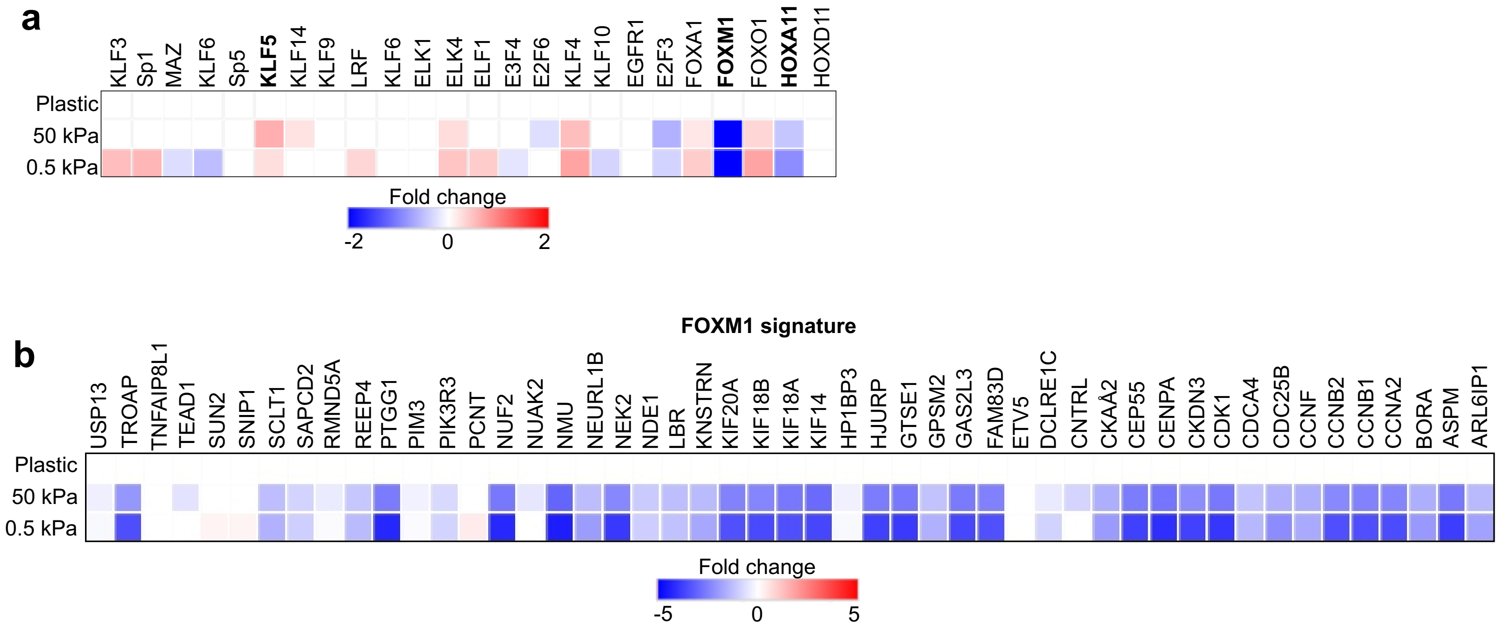

Supplementary figure S2. FOXM1 and its target genes are downregulated in lower ECM stiffness. a) A heatmap showing the RNA-seq derived fold changes of genes selected based on the motif enrichment analysis. Transcription factors FOXM1 and HOXA1 are downregulated in response to decreased ECM stiffness while transcriptional activator KLF5 is upregulated. b) An inspection of FOXM1 signature gene set shows that a number of these genes are downregulated in response to decrease in matrix stiffness. The fold changes are shown as a heatmap.
