# Supplementary material for: Matrix stiffness modulates androgen response genes and chromatin state in prostate cancer
Source: NAR Cancer. 2025 Mar 20;7(1):zcaf010. doi: 10.1093/narcan/zcaf010 (PMC11923743; doi:10.1093/narcan/zcaf010)
Supplement: zcaf010_Supplemental_Files [file zcaf010_supplemental_files.zip › supfig3.pdf]

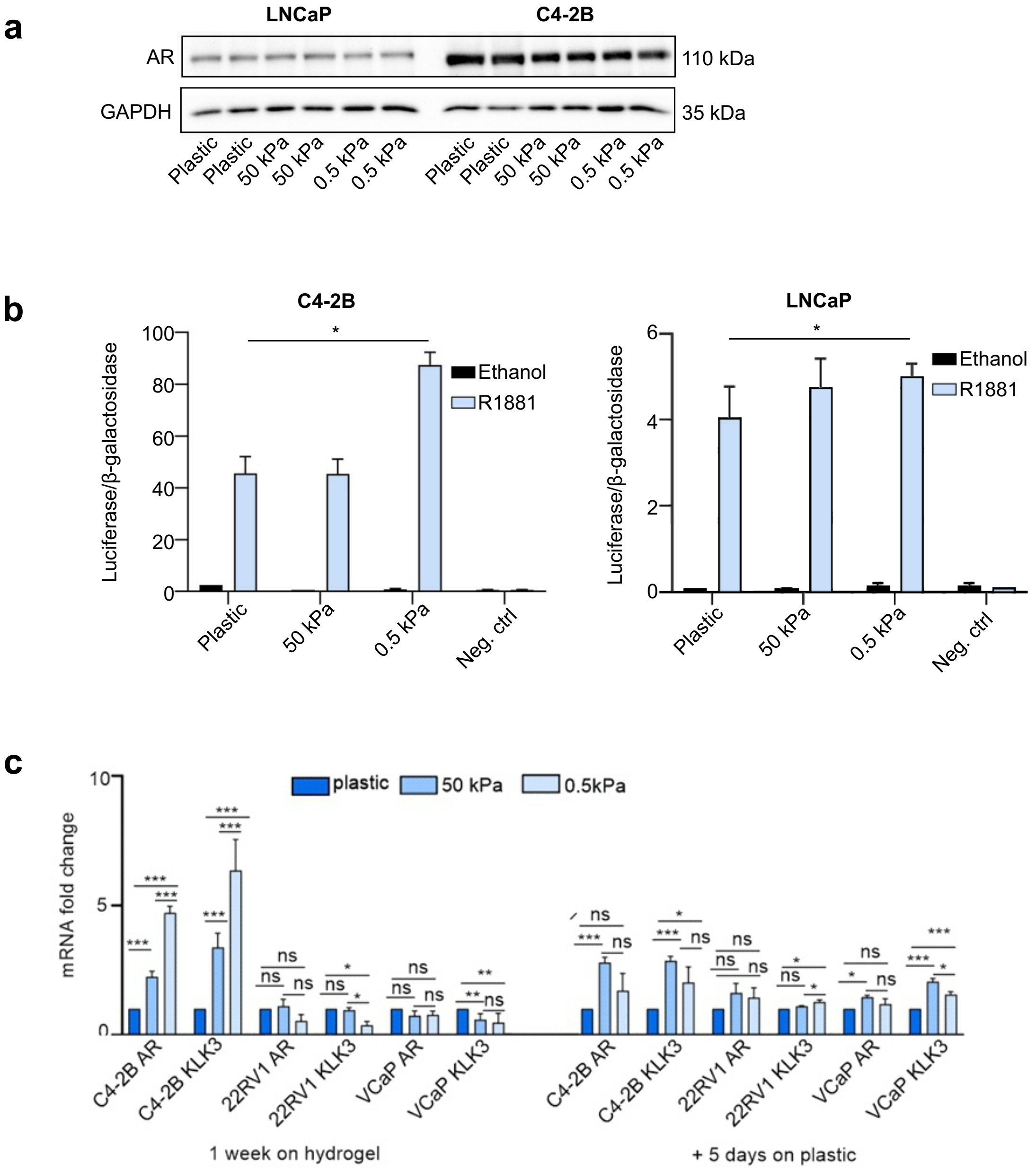

Supplementary Figure S3. a) Western blot analysis of AR protein levels in LNCaP and C4-2B cells grown in either 0.5 kPa hydrogel, 50 kPa hydrogel or plastic plate show that no significant change in AR expression is observed in response to change in ECM stiffness. b) Luciferase reporter assay was used to analyze AR activity in different ECM stiffnesses in C4-2B and LNCaP cell lines. AR binding in response to synthetic androgen R1881 (metribolone) significantly increased on 0.5 kPa hydrogel when compared to plastic. c) C4-2B, 22Rv1 and VCaP cells were grown for one week either on plastic or on 50 kPa or 0.5 kPa hydrogels and then cells were transferred to plastic plates for additional 5 days of growth. No significant induction in the expression of AR and its target was recognized with qRT-PCR in VCaP and 22RV1.
