# Supplementary material for: Matrix stiffness modulates androgen response genes and chromatin state in prostate cancer
Source: NAR Cancer. 2025 Mar 20;7(1):zcaf010. doi: 10.1093/narcan/zcaf010 (PMC11923743; doi:10.1093/narcan/zcaf010)
Supplement: zcaf010_Supplemental_Files [file zcaf010_supplemental_files.zip › supfig4.pdf]

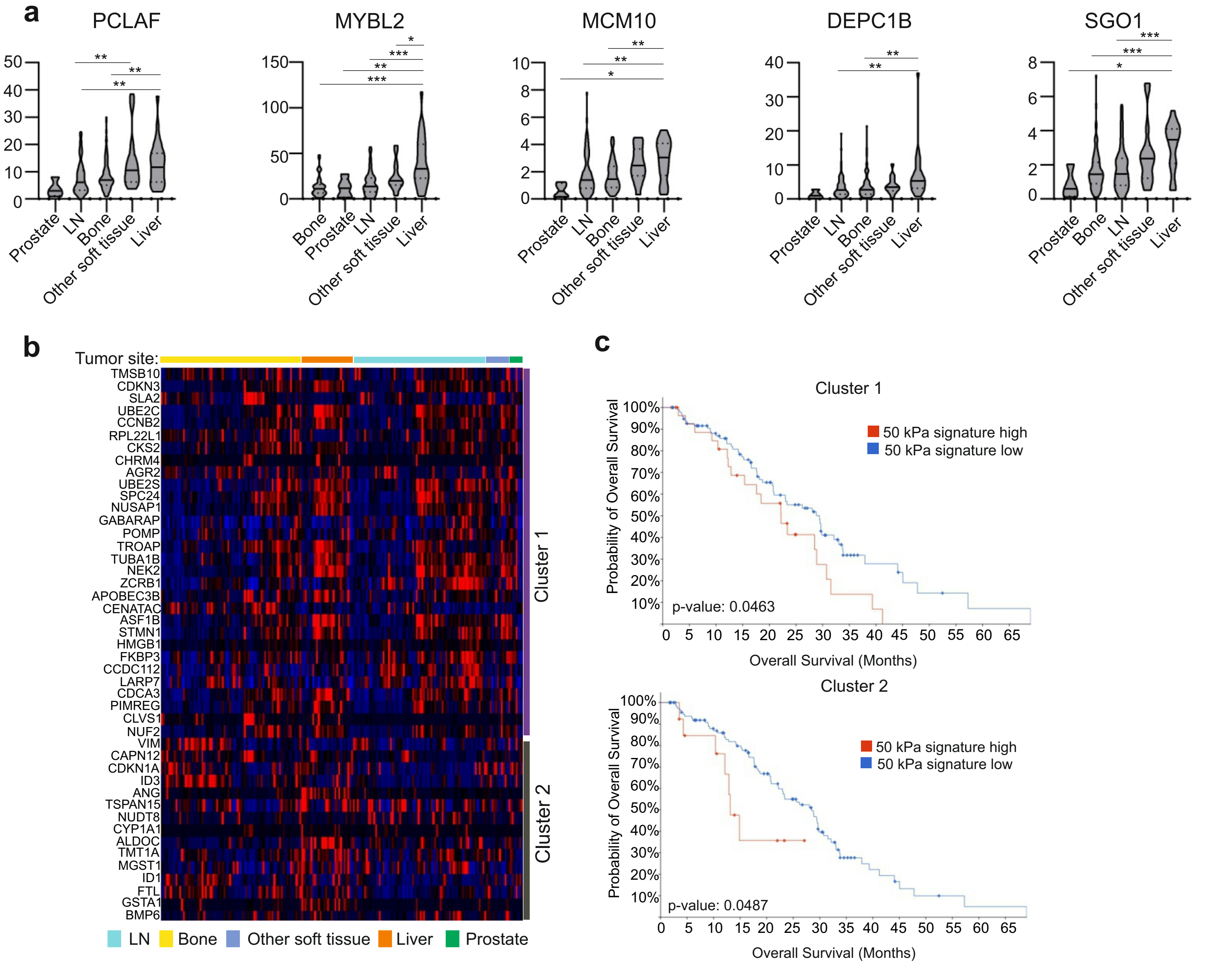

Supplementary Figure S4. a) Expression of five plastic signature genes in prostate, lymph node, bone, other soft tissue and liver metastases in SUC2 metastatic prostate adenocarcinoma patient dataset. Expression of the genes are highest in liver metastases b) Heatmap showing the expression of 50 kPa signature genes on metastatic patient tumor samples from lymph node (turquoise), bone (yellow), other soft tissue (violet), liver (orange) and in prostate (green). c) The overall survival of patients with high expression 50 kPa signature genes is low in both gene clusters.
